# 895. Multisite Derivation and Validation of Machine Learning Models to Predict Severe Influenza Outcomes in Emergency Department Patients

**DOI:** 10.1093/ofid/ofad500.940

**Published:** 2023-11-27

**Authors:** Jeremiah S Hinson, Xihan Zhao, Katherine Z J Fenstermacher, Richard E Rothman, Oluwakemi Badaki-Makun, Scott Levin, Eili Klein

**Affiliations:** Johns Hopkins University School of Medicine, Baltimore, Maryland; Johns Hopkins University, Baltimore, Maryland; Johns Hopkins University, Baltimore, Maryland; Johns Hopkins University, Baltimore, Maryland; Johns Hopkins School of Medicine, Baltimore, Maryland; Beckman Coulter, Washington, District of Columbia; Johns Hopkins School of Medicine, Baltimore, Maryland

## Abstract

**Background:**

Millions of Americans are infected by influenza each year. Most experience mild and self-limited illness but a minority progress to severe disease and death. Emergency departments (ED) serve as a frontline for the US health system and must distinguish those at risk for clinical deterioration from those who can be safely discharged to the community.

**Methods:**

We developed random forest machine learning (ML) models to estimate risk for two severe outcomes in ED patients with influenza: need for inpatient care within 72 hours and need for critical care within 24 hours. Both were defined by multidisciplinary consensus using markers of cardiovascular and respiratory dysfunction captured in the electronic health record (EHR). Predictor data were limited to those recorded prior to ED disposition decision (i.e., discharge, hospitalize) and included patient demographics, ED complaint, active medical problems, vital signs and trends, supplemental oxygen use and selected laboratory results. Our cohort was comprised of adult patients who visited one of five EDs in our university health system between 01/01/2017 and 05/18/2022. A development cohort comprised of encounters that occurred before December 2019 was used for model derivation and 10-fold out of sample validation; models were externally validated in the cohort of encounters that occurred afterward.

**Results:**

Among 8,032 patients with confirmed influenza, incidence of critical care needs at 24 hours was 6.2% (n = 502) and incidence of inpatient care needs at 72 hours was 16.1% (n = 1295). The most common reasons for ED visit were symptoms of upper or lower respiratory tract infection, fever, and shortness of breath. During external validation, models exhibited excellent predictive performance with AUCs of 0.90 (0.87 - 0.94) for critical and 0.89 (95% CI 0.86 -0.92) for inpatient care needs; both were well-calibrated (Brier scores 0.025 and 0.036). Important predictors of severe disease included shortness of breath, increasing respiratory rate and a high number of comorbid diseases.

Predictive Model Performance During External Validation.

**Figure:**
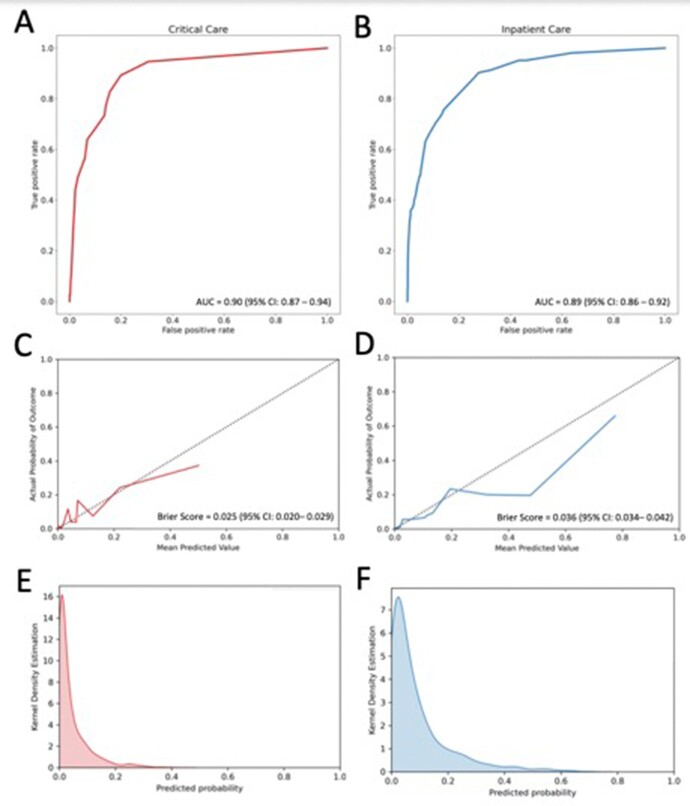

Random forest models predicted risk for critical care needs within 24 hours (red) and inpatient care needs within 72 hours (blue). Overall predictive performance is shown using receiver operating characteristic curves with area under the curve (AUC) in panels A and B; model calibration curves with Brier scores are shown in C and D; predicted probability distributions are shown with kernel density estimation plots in E and F.

**Conclusion:**

These findings demonstrate that EHR data and ML can be used to risk-stratify patients with influenza early in their hospital course. An important future aim is to integrate ML-driven predictions into clinical decision support at the point of care.

**Disclosures:**

**Jeremiah S. Hinson, MD, PhD**, Beckman Coulter: Advisor/Consultant|Beckman Coulter: Grant/Research Support|Beckman Coulter: TriageGO **Katherine ZJ Fenstermacher, PhD**, Cepheid: Grant/Research Support **Richard E. Rothman, MD, PhD**, CEPHEID: Advisor/Consultant|Cepheid: Grant/Research Support|Danaher: Grant/Research Support **Oluwakemi Badaki-Makun, MD, PhD**, Beckman Coulter: Grant/Research Support **Scott Levin, PhD**, Beckman Coulter: Licenses|Beckman Coulter: Employment|Beckman Coulter: Stocks/Bonds

